# The Young Adult Sleep model: an evolving causal loop diagram of mental health dynamics

**DOI:** 10.1186/s12916-026-04738-7

**Published:** 2026-03-16

**Authors:** Jeroen F. Uleman, Rushd F. M. Al-Shama, Adrian G. Zucco, Jette Echterhoff, Maartje Luijten, Maaike Verhagen, Jana Vyrastekova, Jorien L. Treur, Robyn E. Wootton, Sicelo Jones, Martin Dresler, Henning J. Drews, Christine Egebjerg, Birgitte R. Kornum, Karien Stronks, Naja Hulvej Rod

**Affiliations:** 1 Copenhagen Health Complexity Center, Department of Public Health, University of Copenhagen, Copenhagen, Denmark; 2 Department of Clinical and Experimental Cardiology and Cardiothoracic Surgery, Heart Center, Amsterdam , UMC Location University of Amsterdam, Amsterdam, The Netherlands; 3 Behavioural Science Institute, Faculty of Social Sciences, Radboud University, Nijmegen, The Netherlands; 4 Institute for Management Research, Radboud University, Nijmegen, The Netherlands; 5 Department of Psychiatry, Amsterdam UMC, University of Amsterdam, Amsterdam, Netherlands; 6 School of Psychological Science, University of Bristol, Bristol, UK; 7 MRC Integrative Epidemiology Unit, University of Bristol, Bristol, UK; 8 Nic Waals Institute, Lovisenberg Diaconal Hospital, Oslo, Norway; 9 PsychGen Centre for Genetic Epidemiology and Mental Health, Norwegian Institute of Public Health, Oslo, Norway; 10 Donders Institute for Brain, Cognition and Behaviour, Radboud University Medical Center, Nijmegen, The Netherlands; 11 Department of Public Health, University of Copenhagen, Copenhagen, Denmark; 12 Department of Neuroscience, Faculty of Health and Medical Sciences, University of Copenhagen, Copenhagen, Denmark; 13 Department of Public and Occupational Health, Amsterdam University Medical Center, University of Amsterdam, Amsterdam, The Netherlands

**Keywords:** Sleep, Mental health, Young Adult, Feedback loop, System, Epidemiology, Depressive symptoms, Natural language processing, Causal loop diagram, Complex

## Abstract

**Background:**

This study introduces the Young Adult Sleep model, a comprehensive causal loop diagram (CLD) developed to explore the dynamic feedback mechanisms underlying sleep problems and affective depressive symptoms in young adults—an urgent public health challenge.

**Methods:**

The CLDs was developed through five asynchronous questionnaire-based assignments completed by a panel of 14 domain experts, two existing CLDs, and targeted reviews of the scientific literature. Natural language processing was used to curate the system variables from questionnaire data.

**Results:**

The CLD integrates extensively interconnected variables across biological, psychological, behavioral, and social domains. It comprises 29 variables and 175 causal connections, forming numerous reinforcing feedback loops that can drive “vicious” cycles, such as the interplay of sleep disturbances and affective depressive symptoms with addictive behaviors like smoking. The experts also identified balancing loops that may counteract these self-reinforcing dynamics. Many loops span multiple domains, underscoring the importance of multidomain interventions and of interdisciplinary research that synthesizes evidence across scientific fields.

**Conclusions:**

The Young Adult Sleep model is an evolving CLD framework that is intended to be further refined as new evidence becomes available. It supports iterative theory development and hypothesis generation, and serves as a foundation for future computational modeling to simulate intervention strategies to address this complex public health problem.

**Supplementary Information:**

The online version contains supplementary material available at 10.1186/s12916-026-04738-7.

## Background

The decline in mental health among young adults over the past two decades has become a significant public health concern [1]. Indeed, mental health disorders are a major contributor to disability-adjusted life years and place a substantial economic burden on global healthcare systems [2]. Depressive symptoms are prominent and increasing components in the manifestation of these mental health problems in young adults [3, 4]. Sleep disturbances are likewise increasingly common at this life stage [5], and they frequently co-occur with affective depressive symptoms, further complicating the trajectory and hindering recovery. This interplay between sleep and depression not only diminishes the quality of life during young adulthood but also elevates long-term risks, including an increased likelihood of comorbid mental health issues [6, 7], cardiovascular diseases [8, 9], dementia [10–12], and premature death [13, 14].

Mounting evidence suggests that disturbed sleep and depressed mood are not simply co-occurring conditions but rather exist in a complex, bidirectional relationship [7, 15–20]. This interplay occurs through diverse pathways, including biological processes, behavioral patterns, and social dynamics. Together, these factors create self-reinforcing feedback loops, where poor sleep can worsen mood, and, in turn, depressed mood can further disrupt sleep. Despite this understanding of the underlying complexity, much of the current literature examines the relationship between sleep and mental health through a predominantly unidirectional and discipline-specific lens, typically focusing on how a single factor, or a limited set of factors, influences another. While these studies are crucial for disentangling the causality of individual relations, this approach risks overlooking the broader system of interacting and mutually reinforcing mechanisms. Consequently, there is a need for a systematic analysis of the feedback loops that can perpetuate the cycle of sleep problems and affective depressive symptoms across scales and domains.

To address such questions, mental health research increasingly draws on complex systems science [21–24], which recognizes feedback loops as essential features of complex problems [25–28]. Network theory has been a major catalyst, proposing that reinforcing feedback loops among symptoms “give rise to the phenomenological manifestation of mental disorders” [29] and motivating extensive research efforts [30]. Network analyses have closely linked insomnia with generalized anxiety and depression [31], connected sleep disturbances with fatigue and appetite [32], and found that difficulty initiating sleep, but not sleep duration, predicts first-onset depression [33]. Building on this foundation, researchers have emphasized incorporating external factors, such as social support, directly into the endogenous system structure to capture how symptoms interact with and feed back into the broader system [22].

To map system-wide structures, graphical tools like causal loop diagrams (CLD) offer a valuable complementary approach [21, 34–37]. CLD can also be computationally simulated as system dynamics models to analyze system behavior over time and evaluate potential interventions through “what-if” scenarios [12, 35, 38, 39]. While several CLD have been developed for mental health problems (e.g., [21, 34, 40–43]), none of these studies specifically addressed the critical role of disturbed sleep in young adult mental ill health, missing the distinct feedback mechanisms that mutually reinforce and sustain these issues across scales and domains.

To fill this gap, we developed the Young Adult Sleep model: a CLD that maps the complex system underlying sleep problems and affective depressive symptoms in young adults, with a focus on reinforcing feedback mechanisms spanning biopsychosocial scales, starting at the individual level. The diagram was constructed using the collective expertise of a domain expert group, informed by a series of assignments and corresponding literature reviews, and refined using natural language processing. We propose the Young Adult Sleep model as an evolving framework for systematically integrating interdisciplinary knowledge on young adult sleep and mental health problems. The model can be refined and updated as new evidence emerges or expanded to incorporate broader societal factors, providing insights to help understand and inform tailored responses to this pressing public health problem.

## Methods

### Causal loop diagram

A CLD is a graphical representation of the presumed structure of a system consisting of variables connected by (causal) connections [37]. These connections are represented as arrows, each with an assumed direction of causality and a positive or negative polarity. A positive polarity (+) means that when one variable increases (or decreases), the connected variable changes in the same direction, or at least reaches a higher (or lower) value than it would have without the change [44]. In contrast, a negative polarity (–) indicates a change in the opposite direction.

A distinctive feature of CLD is the presence of feedback loops, which can be either reinforcing or balancing, depending on the polarities of the connections. If the loop contains an odd number of negative polarities, it is balancing; otherwise, it is reinforcing. Reinforcing loops have a strengthening influence, leading to instability and exponential growth. In contrast, balancing loops have a corrective influence and tend toward equilibrium. Identifying feedback loops and determining whether they are reinforcing or balancing is essential, as they often underlie nonlinear dynamics that amplify small changes and can potentially drive emergent challenges, such as the current mental health crisis.

### Causal loop diagram development steps

To provide a systematic and comprehensive overview of the key variables and their interconnections involved in sleep problems and affective depressive symptoms in young adults, we combined three sources of evidence. These sources include expert knowledge from a group of 15 domain researchers, supplementary reviews of the scientific literature, and two existing CLD [21, 40].

Our approach to developing the CLD consisted of the following three phases: (1) define the dynamic problem, (2) identify key system variables, and (3) map the system [37]. Each step is explained in more detail below. The modeling team responsible for problem conceptualization, assignment formulation, and assessment of the experts’ responses consisted of JFU (the main modeler) and RAS, KS, and NHR (who also served as experts). The members of the modeling team were involved in conceptualizing most, but not necessarily all, steps.

#### Define the problem

**Fig. 1:**
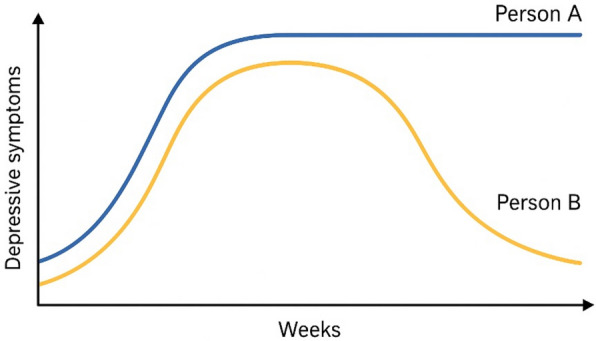
Reference mode: the dynamics of two hypothetical persons’ depressive symptoms over 1 year

The modeling team initiated the construction of the CLD by formulating a dynamic problem that the diagram should help explain. Plots of such dynamic problems are often called “reference modes” [45]. The reference mode for our problem of interest is shown in Fig. 1, where trajectories of two hypothetical individuals experiencing sleep problems and other depressive symptoms are depicted over 1 year. These trajectories are assumed to be symptomatic of the system of biopsychosocial factors underlying these reference modes. The CLD’s focus is on the dynamics that can trap individuals in a vicious cycle. However, the reference mode shows that this cycle can eventually slow and reverse, implying the presence of balancing mechanisms.

The modeling team then defined model boundaries. Such boundaries enable greater precision in the research questions and the insights that can be drawn from the CLD [37]. To reflect the problem’s multiscale nature, the modeling team conceptualized it as occurring across four domains: biological, psychological, behavioral, and social. These domains match one of the existing CLDs [40]. On the other hand, the modeling team restricted the CLD to individual-level variables, concentrating on aspects such as an individual’s experience of their social relationships. Integrating a focus on the person’s interaction with their broader environment, e.g., climate and society at large, would be an obvious next step, but is beyond the scope of the current project. Finally, the modeling team defined the target population as young adults aged 18–40 [4] to encompass a broad range of life transitions relevant to sleep and mental health, including career stress, forming long-term partnerships, and raising young children. To capture the dynamics of symptom onset, maintenance, and resolution rather than on long-standing conditions, we focused on young adults without preexisting psychiatric conditions.

Once the dynamic problem was formulated and model boundaries clarified, the modeling team invited a multidisciplinary international group of 14 domain experts (all scientific researchers, including two medical doctors) to participate in developing the CLD through a series of asynchronous, individual assignments. The workflow of these assignments is summarized in Additional file 1: Figure S1. The modeling team aimed to ensure broad coverage of the four domains. Consequently, the expert group included researchers with diverse expertise, such as sleep biology, well-being, genetic epidemiology, substance use, public health, immunology, general medicine, and sociology. Detailed information about the composition and expertise of the expert group is available in Additional file 2.

#### Key system variables

In assignment 1, the experts were asked to create a list of variables they considered critical for understanding the reference mode (Fig. 1) and to provide a motivation for each variable in the process. The experts were instructed to focus on their area of expertise and to propose variables within the predefined model boundaries. Combined, the experts proposed 180 variables, of which 151 were unique (Additional file 1). Out of these unique variables, some represented the same constructs but were formulated slightly differently (e.g., “Physical activity” and “Exercise/physical activity”). Additionally, some variables differed but were conceptually similar enough to be considered part of a broader overarching construct, such as “Cortisol,” “Biological stress,” “Physiological stress response,” and “HPA axis activity.” Therefore, we next went through a process of merging such variables.

To combine and aggregate conceptually similar variables, we used natural language processing to map the variables proposed by the experts into a common vector space using pre-trained word embeddings. We first utilized GloVe embeddings trained on the English Wikipedia (glove-wiki-gigaword-100) [46], implemented in Python 3.11 using the Gensim library [47]. Each variable was represented by the average of its word embeddings (after removing stop words like “a,” “the,” “is,” and “are”). We then calculated pairwise cosine distances between the variables to quantify semantic similarity. Because the total number of unique variables proposed by the experts was relatively small, the modeling team could exhaustively review the results. Variable pairs with high cosine similarities (> 0.8; although smaller similarities were also explored) were flagged as potentially similar, and then manually combined into broader, conceptually meaningful categories by the modeling team. For instance, variables such as “Immune system/common infections,” “Inflammation,” “Brain inflammation,” and “Infections/Inflammation” were synthesized into an overarching variable, *Chronic inflammation*, which the experts subsequently accepted. In this phase, the similarity measures provided guidance, but the final synthesis relied on expert assessment.

To validate these groupings, we then employed a sentence-transformer model (paraphrase-multilingual-mpnet-base-v2) [48], which was the highest-performing model in a recent evaluation of approaches for merging CLD variables [49], using the sentence-transformers Python package [50]. We again utilized cosine similarities based on these embeddings and applied agglomerative hierarchical clustering using the scikit-learn Python package [51]. A similarity threshold for cluster formation of 0.58 was selected using the kneedle algorithm (see Additional file 1) [52] at the point where a lower cosine similarity cut-off would lead to a marked increase in pairs that would be considered similar. We used average linkage to reduce the influence of outliers and promote balanced cluster formation. An overview of the resulting clusters and their correspondence to the final CLD variables is provided in Additional file 1.

In addition to proposing variables, assignment 1 required the experts to rate the relevance/importance of the proposed variables (1–3, with three meaning “high importance”). We used these ratings to prioritize which variables would make it into the final list of key variables for the CLD, using a cut-off of 3. That is, if one of the experts proposed a variable and rated it as highly important or three experts proposed it with “low importance,” the variable was included in the key variable list. For variables mentioned multiple times or combined in an overarching variable like *Body fat*, we summed up the ratings each expert gave (e.g., two experts rated *Body fat* as two and another as three, so the overall score was seven). Finally, 18 variables, including “synaptic pruning” and “accidents,” were excluded because they were proposed only once with a low-importance rating and did not fall under a broader variable category.

After creating the list of key variables, the modeling team proposed definitions for each variable based on the expert input in assignment 1. This list was then sent back to the experts in assignment 2, after which several further changes were made to both the definitions and the variables themselves. For instance, “substance use” was split into *alcohol use* and *cannabis use*, while other substances like cocaine were considered insufficiently prevalent (< 5%) to justify individual inclusion in the CLD.

#### Map the system variables

To go from a list of key variables to a CLD that also maps their connections, the modeling team first integrated the connections from two existing CLD [21, 40]. The CLD by Wittenborn et al. [21] focused on adult depression and was primarily derived from a literature review and underwent critique by a panel of five domain experts. The CLD by Uleman et al. [40] was centered around depressive symptoms in young adults in response to a stressor and was formulated through collaborative group model building sessions involving four domain experts. The group model building was supplemented by a literature review by the same experts and a causal discovery analysis. Three of these domain experts also served as experts in the current project (MV, ML, JV).

The modeling team derived connections from these two diagrams to add to the present CLD based on the key variable list. To achieve this, we first mapped variables from the CLD by Wittenborn et al. [21] to the variables from our CLD (Additional file 1). For instance, “Interpersonal relationship quality” from Wittenborn et al. [21] was mapped onto *social support*. Next, we assessed the supporting literature for the connections in the existing CLD. This was important because, while Uleman et al. [40] focused on the same target population (i.e., young adults), Wittenborn et al. [21] focused on adults in general. Since the causes of depression may differ between young and older adults [53], we included the connection only if the proposed literature’s context matched our CLD’s. If not (e.g., studies focusing on older adults), the modeling team conducted a new literature review to find support for it. These links were then proposed to the domain expert group in the next assignment.

In assignment 3, the domain experts were involved in identifying the remaining connections between variables in the CLD. The experts were provided with an interactive visualization of the CLD, allowing them to assess the key variables and the connections between the existing CLD, with supporting scientific literature, if any. The experts were asked to spend approximately 2 hours filling out a spreadsheet, focusing on their area of expertise. In this file, the experts were first asked to consider the connections from the existing CLD and whether they believed these were not just causal but also direct (i.e., not mediated by any of the other variables in our new CLD). If not, they could suggest their removal or propose an indirect path. After reflecting on the connections from the existing CLD, the experts were asked to propose new connections and then add them to the spreadsheet with their corresponding polarities (“ + ” or “-”).

### CLD annotations: scientific literature and intermediary mechanisms

To support the connections they proposed, the experts were also asked in assignment 3 to provide one or more references to the scientific literature based on a (non-systematic) literature review focused on their area of expertise. As a guideline, the experts were asked to focus on studies relating to the CLD’s target population and on studies reporting interventions (e.g., a randomized controlled trial, if available), well-controlled associations (ideally in a longitudinal study), or plausible underlying mechanisms (e.g., a comprehensive narrative review). When the experts did not provide supporting literature (e.g., due to time constraints), the modeling team conducted a literature review instead.

Additionally, in assignment 3, the experts were asked to provide an explanation for each proposed connection to assess the plausibility of its underlying mechanism. This was important because empirical evidence of causality does not necessarily justify a *direct* connection. Direct connections in the CLD were only retained if the hypothesized underlying mechanisms were not already captured by other CLD variables [35]. To supplement the formulation of plausible underlying mechanisms for connections, the modeling team used a large language model (LLM) (GPT-4 Turbo) to generate candidate mechanisms when expert suggestions were lacking. Although LLM often struggle to distinguish direct from indirect causal effects, they can be effective at suggesting mechanistic explanations for putative direct connections [54] and are increasingly used as a critical thinking tool in the development of systems models [55].

After formulating one or more plausible underlying mechanism(s) for a given connection, one of the following three actions was proposed by the modeling team to the expert group in assignment 4. (1) If at least one identified intermediary mechanism was not a CLD variable, retain the direct connection. (2) If the identified mechanisms were already in the CLD, remove the direct connection and replace it with an indirect path through the relevant CLD variable. (3) If no plausible mechanism could be identified, remove the direct connection. For instance, a proposed connection between *loneliness* and *physical health* was reassessed after the LLM suggested that the effect would be primarily mediated through stress-related pathways and health behaviors, such as physical inactivity and poor diet, which were already included as CLD variables. On the other hand, discrimination was considered a plausible underlying mechanism between *ethnic minority background* and *socioeconomic status*, making a direct connection likely, as discrimination is not a separate variable in the CLD. The modeling team also proposed adding connections when intermediary mechanisms were deemed relevant for multiple connections. In the assignment, the experts then accepted or adjusted these proposed underlying mechanisms and suggested additional connections as needed. Additional revisions were made during this process, after which the revised CLD was circulated in interactive format for further modifications and approval.

### Balancing feedback loops

Because all the loops identified by the experts in assignments 1–4 were reinforcing, the CLD developed up to this stage was focused on unraveling the factors that entangle young adults in these issues rather than mechanisms of recovery. While this problem-oriented focus provides the foundation for understanding escalation processes, a systematic exploration of balancing loops was needed to identify mechanisms that could stabilize the system.

In assignment 5, the experts were therefore each asked to consider 1–3 endogenous variables (excluding exogenous variables without incoming connections). For each variable, the experts were instructed to consider which mechanisms could prevent the theoretically unlimited escalation implied by the reinforcing loops. For example, a reinforcing loop involving *socioeconomic status* may operate as follows: an initial decline in income could lead to sleep and mental health problems, which in turn might reinforce further income decline due to reduced job performance. Without intervention by a balancing mechanism, this dynamic would theoretically continue until complete income loss occurs. However, social security systems can act as a balancing mechanism by providing financial support to those with low incomes, thereby limiting further decline and stabilizing the system.

The experts were asked to provide such balancing loops for each of their assigned variables. Although balancing loops are not inherently beneficial in all contexts, in this assignment, they were intentionally defined as beneficial mechanisms that could counteract problematic reinforcing dynamics. The modeling team assessed all proposed loops and engaged with relevant experts when modifications were deemed necessary. Additionally, the experts were asked one final time to review and refine the explanations and literature references for the incoming connections to their assigned CLD variables in assignment 5.

## Results

### Variables

The final CLD the domain experts agreed upon encompasses 29 variables, distributed as follows: five in the biological domain, seven in the psychological domain, nine in the behavioral domain, and eight in the social domain. An overview of the variables and their respective definitions is provided in Additional file 2.

### Connections

**Fig. 2:**
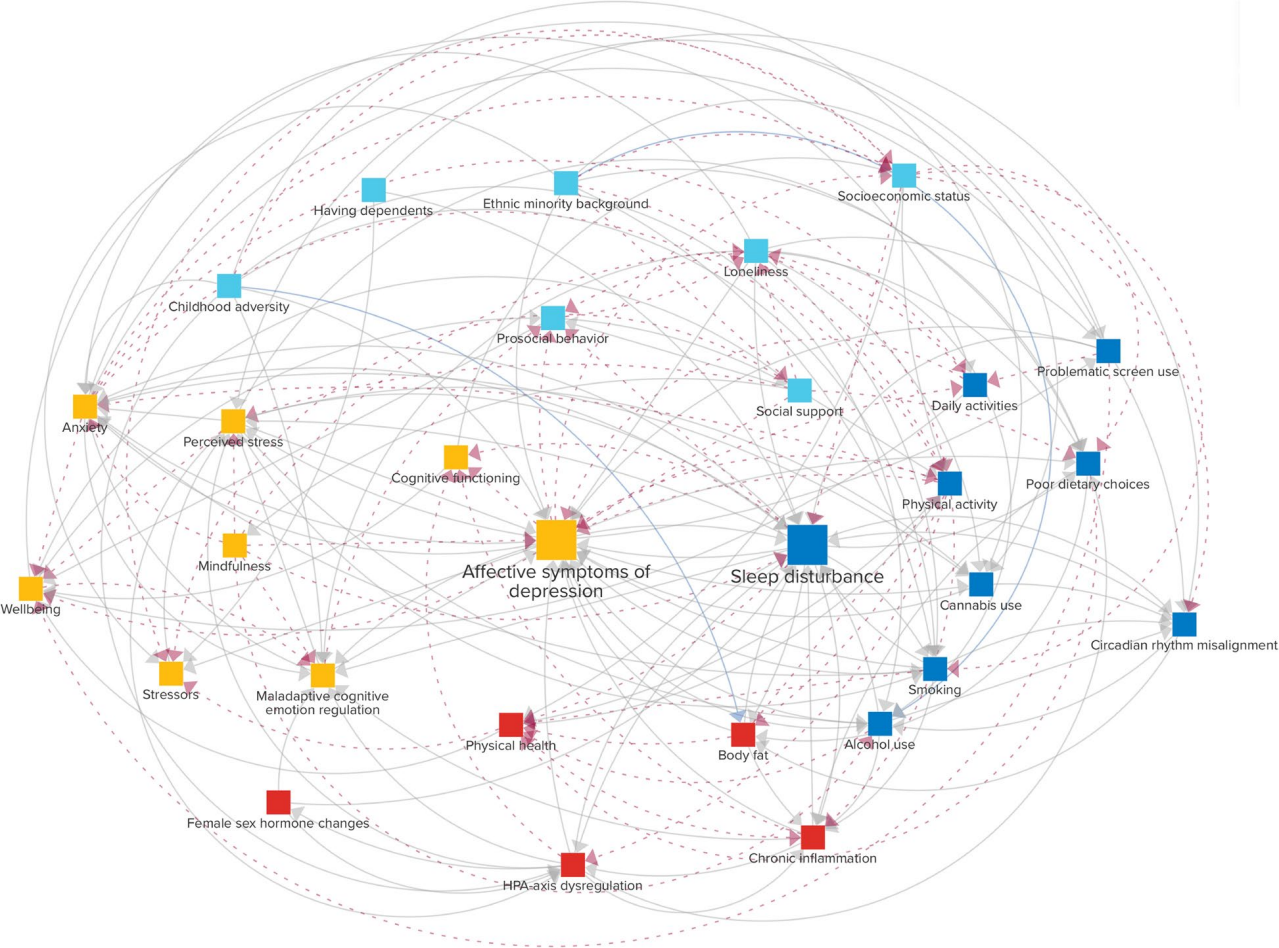
Causal loop diagram of sleep problems and affective depressive symptoms in young adults with biological (red), behavioral (dark blue), psychological (yellow), and social (light blue) levels. Solid gray connections have positive polarity, whereas dashed red connections have negative polarity. Two connections (solid blue) have ambiguous polarity. An interactive version of the diagram is available here https://doi.org/10.17605/OSF.IO/HAV3K

The CLD representing the self-reinforcing system identified in assignments 1–4 comprises 175 proposed connections. Of these, the experts incorporated 60 connections from the two existing CLD: 29 from Uleman et al. [40] and 31 from Wittenborn et al. [21]. The remaining 115 connections came solely from the expert group. The CLD is depicted in Fig. 2. An interactive version of the diagram can be found here 10.17605/OSF.IO/HAV3K. Out of the connections, 58 have negative polarity (dashed red arrows), 115 have positive polarity (solid gray arrows), and two have an ambiguous polarity (blue, solid arrows). In particular, *childhood adversity* can both result in higher and lower *body fat* [56], and *ethnic minority background* can lead to lower *alcohol use* [57] but, in the case of perceived discrimination, can also increase it [58]. Every connection is supported by an explanation and one or more references to scientific literature provided by the experts from their fields of expertise. These explanations and references can be found by clicking on a connection in the interactive version of the CLD. The diagram includes three “exogenous” variables without incoming connections, delimiting the system boundaries. These variables are *childhood adversity*, *ethnic minority background*, and *having dependents*. The other variables are “endogenous,” meaning they both influence—and are influenced by—other variables within the system.

Some of the connections proposed by the experts overlapped. The connection *problematic screen use* → *sleep disturbance* was proposed by five experts, whereas four experts proposed *circadian rhythm disruption* → *sleep disturbance*. Seven connections, including *anxiety* → *affective symptoms of depression*, were proposed by three experts; 26 connections, like *sleep disturbance* → *poor physical health*, were proposed by two experts; and the remaining 102 connections were each proposed by only one expert. The limited overlap is not surprising given that the group was selected to represent varying areas of expertise and thus focused on different aspects of the model.

### Reinforcing feedback loops

**Fig. 3:**
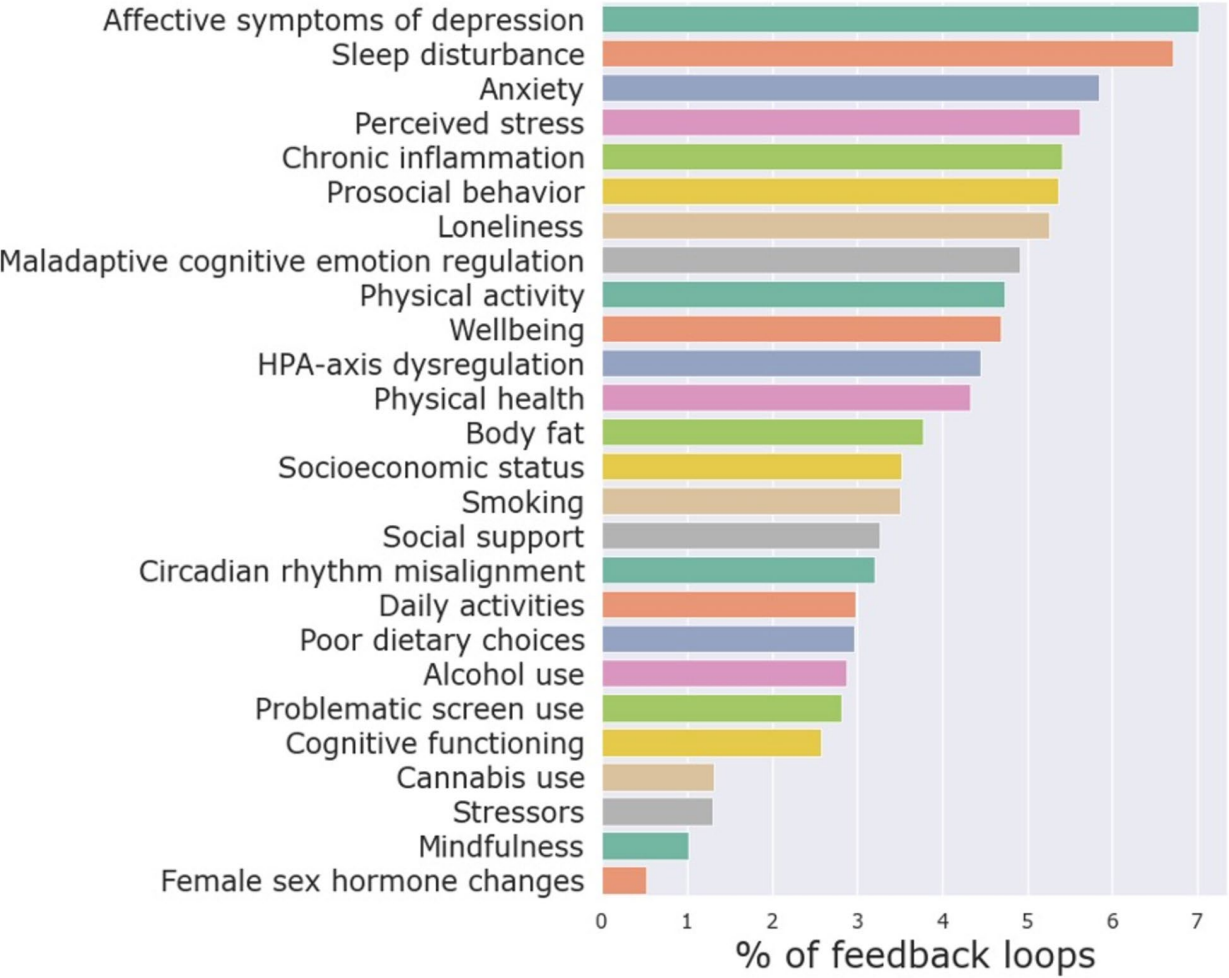
The percentage of feedback loops each variable is part of, considering feedback loops with up to 14 variables. The considered loop sizes were iteratively increased up to length 14, at which point the top 10 ranking had stabilized across two consecutive iterations (lengths 12 and 13), and further expansion sharply increased computational cost. The three exogenous variables are not shown as they do not have any incoming connections and can thus not be part of any feedback loops

The CLD in Fig. 2 consists solely of reinforcing feedback loops: 30 loops with two variables, 113 loops with three variables, 462 loops with four variables, and 1815 loops with five variables each. There are many more feedback loops consisting of larger numbers of variables. To assess which variables may be most impactful on the reinforcing feedback structure, the percentage of feedback loops (with a maximum length of 14) in which each variable participates is shown in Fig. 3. Unsurprisingly, given the focus of the CLD, *affective symptoms of depression* (7.0%) and *sleep disturbance* (6.7%) are relatively part of the most feedback loops. After these, *anxiety* (5.8%), *perceived stress* (5.6%), *chronic inflammation* (5.4%), *prosocial behavior* (5.4%), *loneliness* (5.3%), *maladaptive cognitive emotion regulation* (4.9%), *physical activity* (4.7%), and *well-being* (4.7%) rank highest, suggesting that they might play a key role in the vicious cycle between sleep and mental health problems in young adults.

Given our focus on the reinforcing mechanisms between *sleep disturbance* and *affective symptoms of depression*, we examined loops that include these two variables in more detail. Since the total number of loops is too large to assess exhaustively, we restrict attention to loops of length 3. Shorter feedback loops may be particularly relevant because, by the data processing inequality [59], each added variable in the loop can only reduce the mutual information between a variable’s original and future state, suggesting that longer loops are more likely to yield weaker and more delayed effects. Thirteen loops consisted of both *sleep disturbance* and *affective symptoms of depression* and a third variable out of the following 10: *anxiety*, *HPA-axis dysregulation*, *chronic inflammation*, *problematic screen use*, *smoking*, *alcohol use*, *perceived stress*, *body fat*, *maladaptive cognitive emotion regulation*, and *socioeconomic status*. Among these, *smoking*, *anxiety*, and *perceived stress* stand out as they form feedback loops with *sleep disturbance* and *affective symptoms of depression* in both directions.

**Fig. 4:**
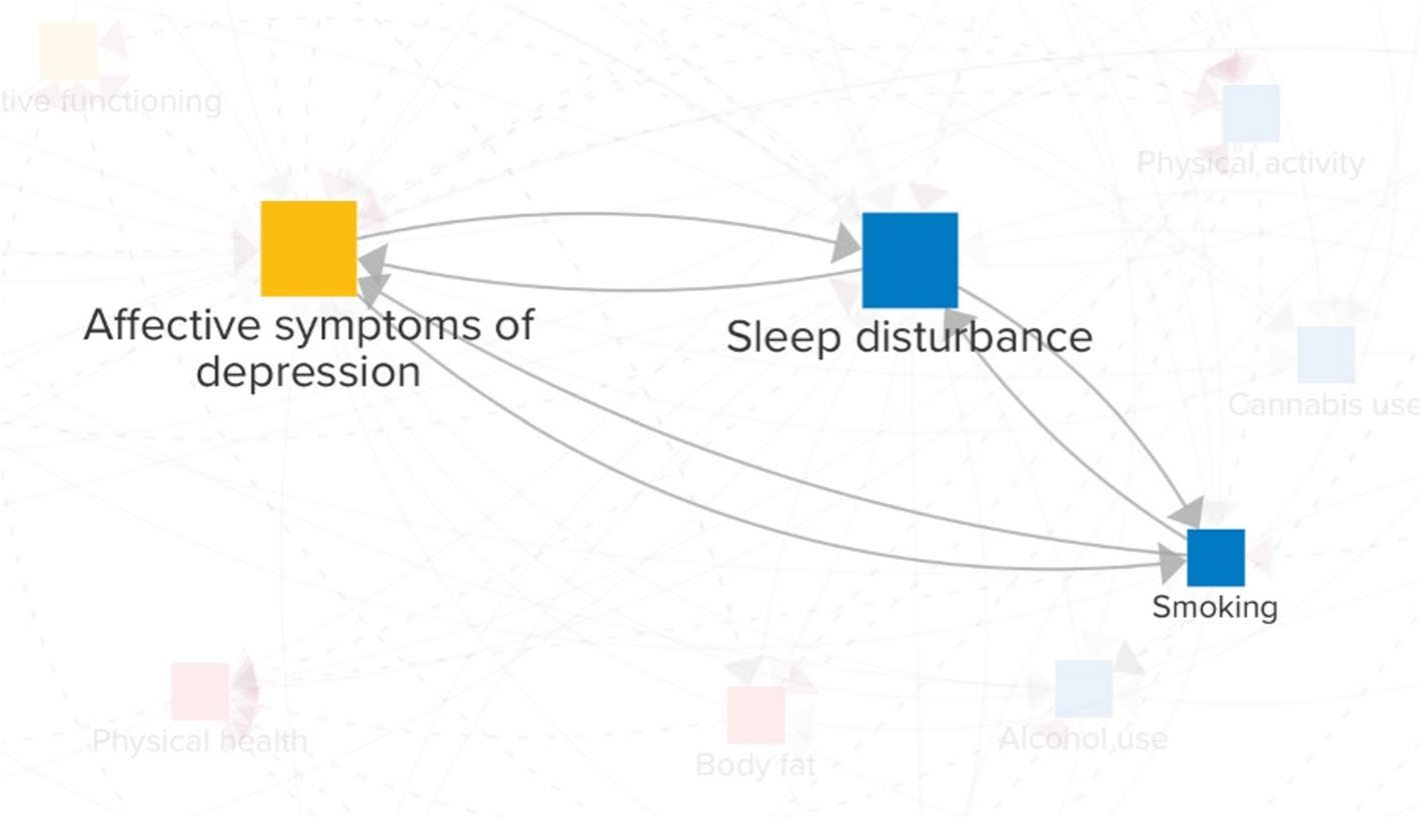
Feedback loops of lengths two and three connecting *sleep disturbance*, *affective symptoms of depression*, and *smoking*

To illustrate, Fig. 4 provides an example for *smoking*, which is linked to a higher risk of developing depressive symptoms [60], potentially due to the neurochemical effects of nicotine. These mood alterations, in turn, can disrupt sleep regulation [61], and this may prompt an increase in cigarette smoking [62] as individuals might use nicotine to counteract daytime sleepiness. However, being a stimulant, nicotine can also hinder one’s ability to fall asleep and degrade sleep quality [63]. This disturbance can further exacerbate depressive symptoms [64]. In response, those suffering from depressive symptoms may increase their smoking frequency [60], possibly as a form of coping. Thus, by influencing the feedback loop in both directions, *smoking* could play a significant role in both triggering and sustaining issues related to both sleep and mental health.

### Within- and cross-scale loops

A key notion is system dynamics that “in complex systems cause and effect are often not closely related in either time or space.” [65] Consequently, when analyzing a CLD, it can be helpful to distinguish within- from cross-scale loops [36]. When all variables in a loop operate within the same scale (e.g., biological processes), the loop is considered “within-scale” while “cross-scale” loops integrate multiple domains. An example of such a cross-scale loop is the abovementioned loop between *affective symptoms of depression* from the psychological domain, *sleep disturbance* from the behavioral domain, and *socioeconomic status* from the social domain. Many more loops like this can be identified in the CLD. These cross-scale loops can represent multidomain interactions that might not be immediately evident from a single scientific domain yet could play pivotal roles in driving the complex dynamics between mental health and sleep.

**Fig. 5:**
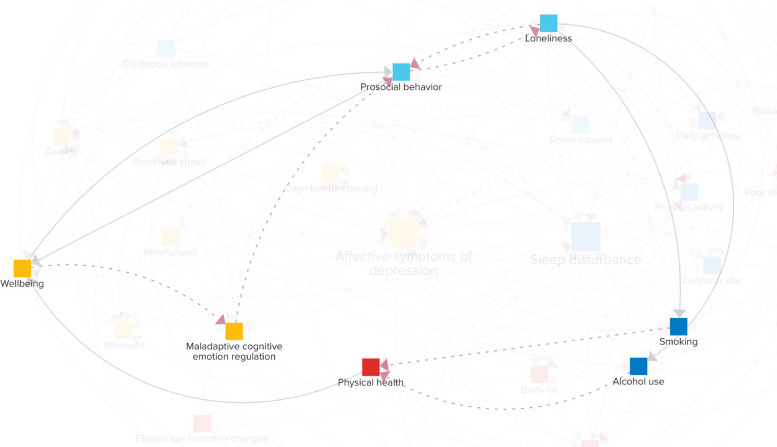
Example of a cross-scale loop involving *well-being*

While the above concentrates on short loops involving *sleep disturbance* and *affective symptoms of depression*, other loops can also be critical for the system’s behavior. For instance, *well-being* integrates multiple domains and is defined to encompass “physical, emotional, social, and spiritual dimensions” (Additional file 2). An illustrative example of a longer cross-scale loop involving *well-being* is depicted in Fig. 5. A lower sense of *well-being* can reduce the use of adaptive emotion regulation strategies [66], such as positive reinterpretation and concentrating on positive things, and increase *maladaptive cognitive emotion regulation*, such as lower impulse control and blaming others. A lack of adaptive emotion regulation strategies can diminish *prosocial behavior* [67], which, in turn, may lead to *loneliness* [68] by impacting the quality and depth of social interactions. Periods of *loneliness* can encourage addictive behaviors like *smoking* and *alcohol use* [69] as a coping mechanism, seeking relief from feelings of isolation. These behaviors can negatively impact one’s health, e.g., affecting cancer risk [70]. *Physical health*, in turn, is an important determinant of *well-being* [66]. Although this loop does not directly impact *affective symptoms of depression* and *sleep disturbance*, it influences several mechanisms that do. Therefore, if this loop is potent, it can indirectly have a strong effect on these issues. A more detailed quantitative analysis using empirical data is necessary to ascertain which loops are primarily responsible for the assumed self-reinforcing dynamics within our system.

### Balancing loops

In addition to the problem-focused CLD with reinforcing loops, the experts identified 66 balancing loops in assignment 5 (see Additional file 2). These balancing loops represent mechanisms that might limit or counteract problematic states within the system, potentially offering pathways toward recovery or stability, and can be seen as an additional layer placed upon the problem-focused, reinforcing CLD. We classified these balancing loops into four categories: homeostatic and resource-limited (14 loops), behavioral (22 loops), clinical (13 loops), and interpersonal (17 loops).

**Fig. 6:**
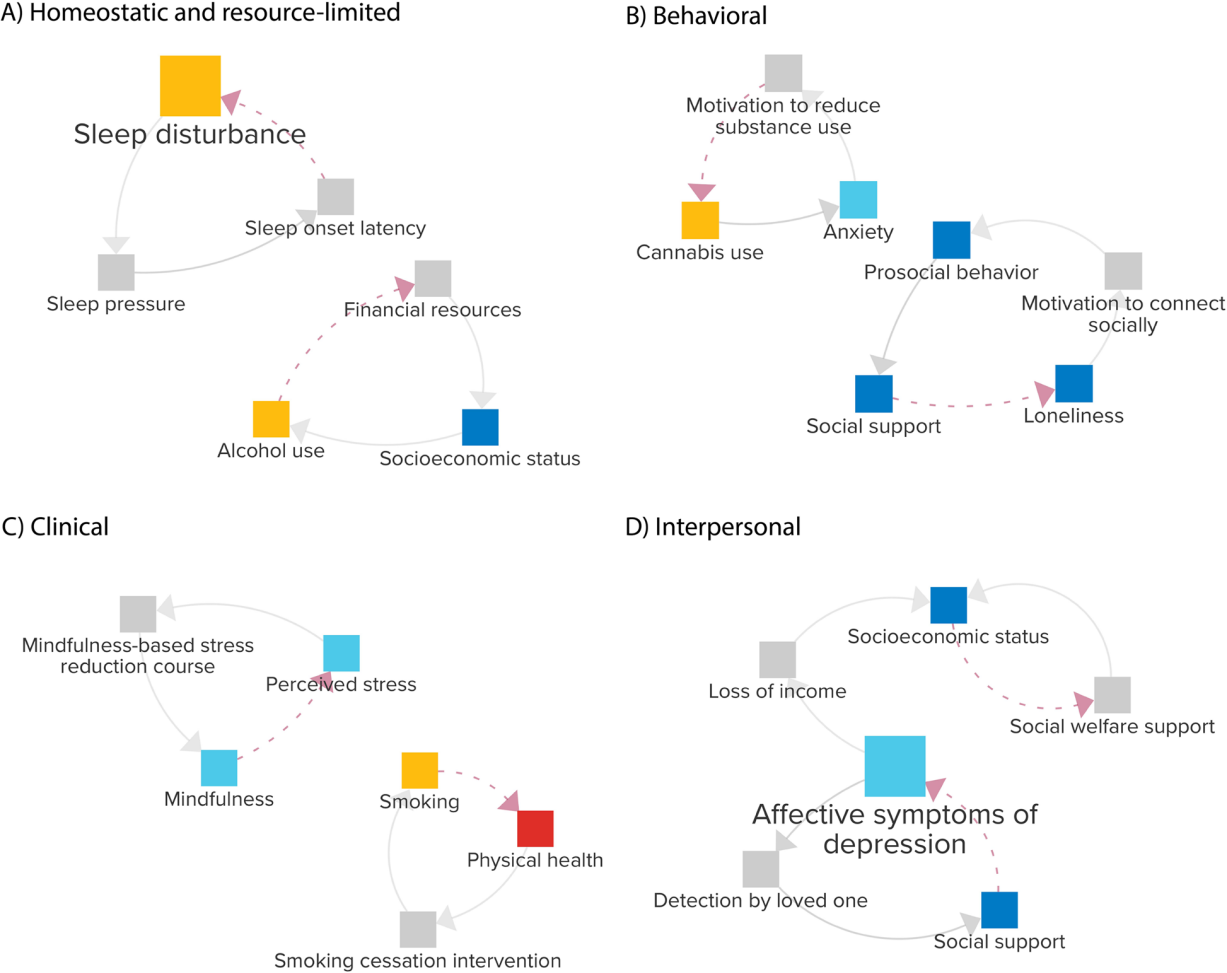
Four-panel figure with examples of balancing loops from each category

Figure 6 provides representative loops for each category, primarily focused on counteracting high-ranked variables in Fig. 3. Homeostatic and resource-limited loops represent automatic biological regulation and external constraints that limit adverse outcomes, such as *sleep disturbance* triggering processes that build sleep pressure, making it easier to fall asleep, or *alcohol use* drawing on financial resources, which may gradually constrain further use. Behavioral loops capture self-initiated attempts to modify lifestyle in response to symptoms like *anxiety*, prompting changes in *cannabis use*, or *loneliness*, motivating engagement in *prosocial behavior*, which fosters *social support* and reduces feelings of *loneliness*. Clinical loops involve treatment-based interventions, including *affective symptoms of depression* leading to engagement in cognitive behavioral therapy or selective serotonin reuptake inhibitors, which reduce further treatment-seeking, or stress prompting enrollment in mindfulness-based stress reduction courses to enhance *mindfulness* and lower *perceived stress*. Finally, interpersonal loops involve external detection of problems and interventions provided by social networks or organizations, such as a loved one noticing *affective symptoms of depression*, offering *social support*, or declining income triggering social welfare support, which stabilizes or improves the individual’s situation. Together, these balancing loops represent diverse mechanisms that might be leveraged for (preventive) interventions, from supporting homeostatic processes to providing guidance on lifestyle change and strengthening institutional support.

### Hypothetical individual narratives

The CLD outlines multiple potential pathways to sleep problems and affective depressive symptoms at the individual level, but these pathways may not equally apply to every young adult, as evidence is derived mostly from group-level averages and, therefore, cannot always be generalized to every individual. Instead, the CLD functions as a meta-diagram that synthesizes diverse individual trajectories into an overarching model, where the importance of each pathway depends on a person’s unique circumstances. To illustrate this, we present two hypothetical case studies corresponding to the reference mode in Fig. 1: person A, a female student adapting to a new social environment; and person B, a male immigrant with a history of *childhood adversity*. These narratives illustrate the variable applicability of the pathways within the CLD across different personal contexts. Each narrative also highlights how balancing loops might lead to symptom stability or resolution.

### Hypothetical person A: student in a new social environment

**Fig. 7:**
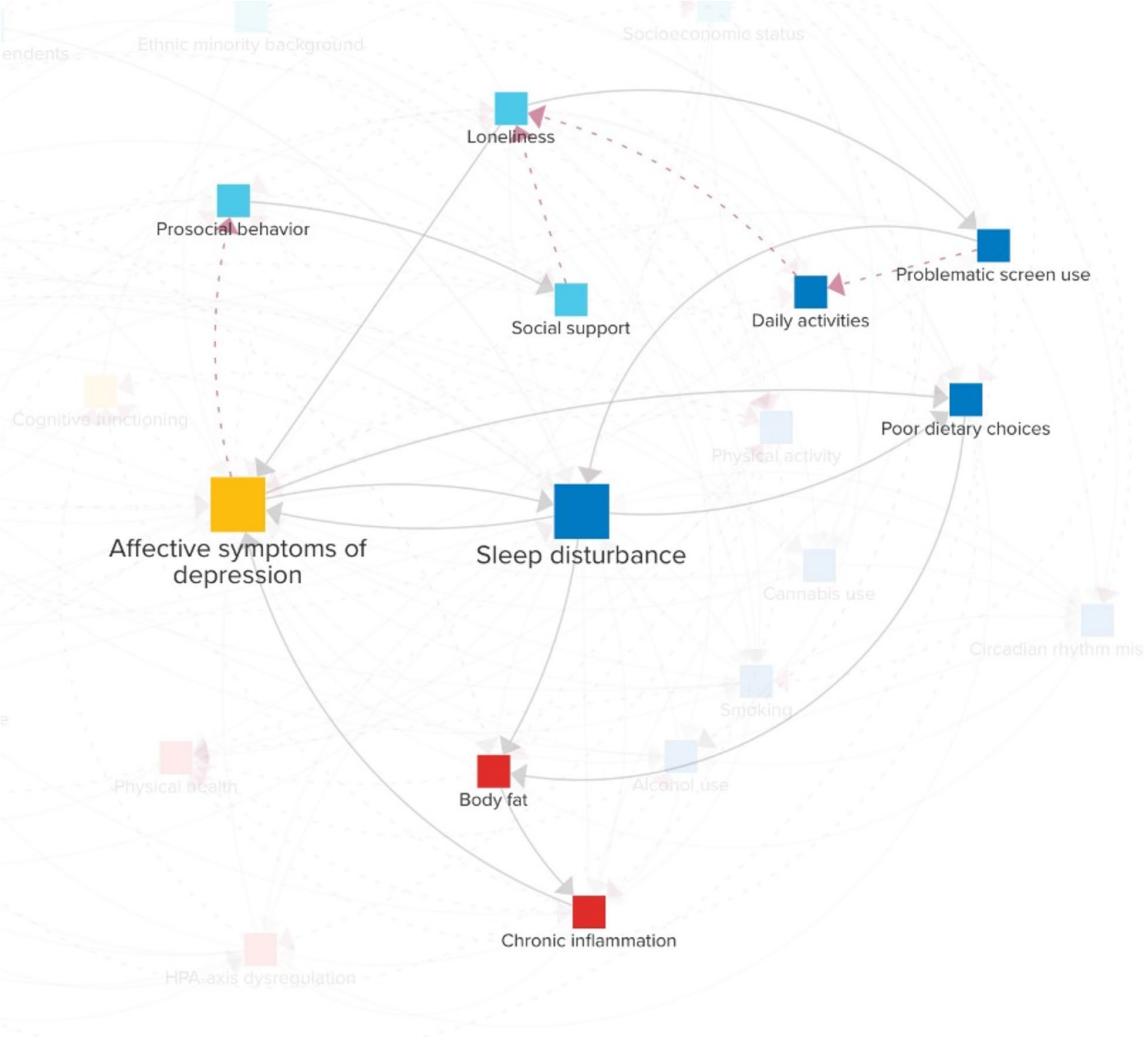
The relevant causal loop diagram pathways for a hypothetical case-scenario of a student in a new social environment (person A)

Figure 7 describes the paths relevant to person A, a young woman in her late teens who recently transitioned to a new school in an unfamiliar city. From the start, person A struggled to adapt to the new environment, where established social groups left her feeling excluded. Although not outright ostracized, she experienced a lack of meaningful social connections, leading to a growing sense of loneliness.

In an attempt to cope, person A began spending increasing amounts of time on social media and streaming platforms. While these activities provided a temporary escape, they did not foster genuine relationships. Instead, excessive screen time reduced her opportunities for face-to-face interaction, further compounding her *loneliness*. Consequently, over the following weeks, person A started to exhibit *affective symptoms of depression*. These symptoms further diminished her sense of belonging and impaired her ability to function socially, causing her to behave less prosocially at school. As a result, the quality of the few friendships she had formed declined, exacerbating her perceived lack of *social support*.

Meanwhile, nighttime screen use began to interfere with her sleep quality, and poor sleep intensified her depressive symptoms, creating a mutually reinforcing cycle of sleep problems and low mood. As her sleep and mental health worsened, person A’s dietary habits also deteriorated. Frequent late-night snacking on calorie-dense, nutrient-poor foods became a coping mechanism for her low mood and fatigue. These dietary changes, combined with the ongoing sleep deficiency, contributed to unhealthy body weight, creating a proinflammatory milieu that further perpetuated the depressive symptoms she was experiencing.

By the end of the school year, person A’s symptoms had reached a critical point. Person A was diagnosed with major depressive disorder and insomnia. Although the diagnosis marked the beginning of professional intervention, including pharmacological treatment that halted symptom progression, her recovery was a long and challenging process due to the deeply entrenched feedback loops driving her condition.

### Hypothetical person B: immigrant exposed to high adversity in childhood

**Fig. 8:**
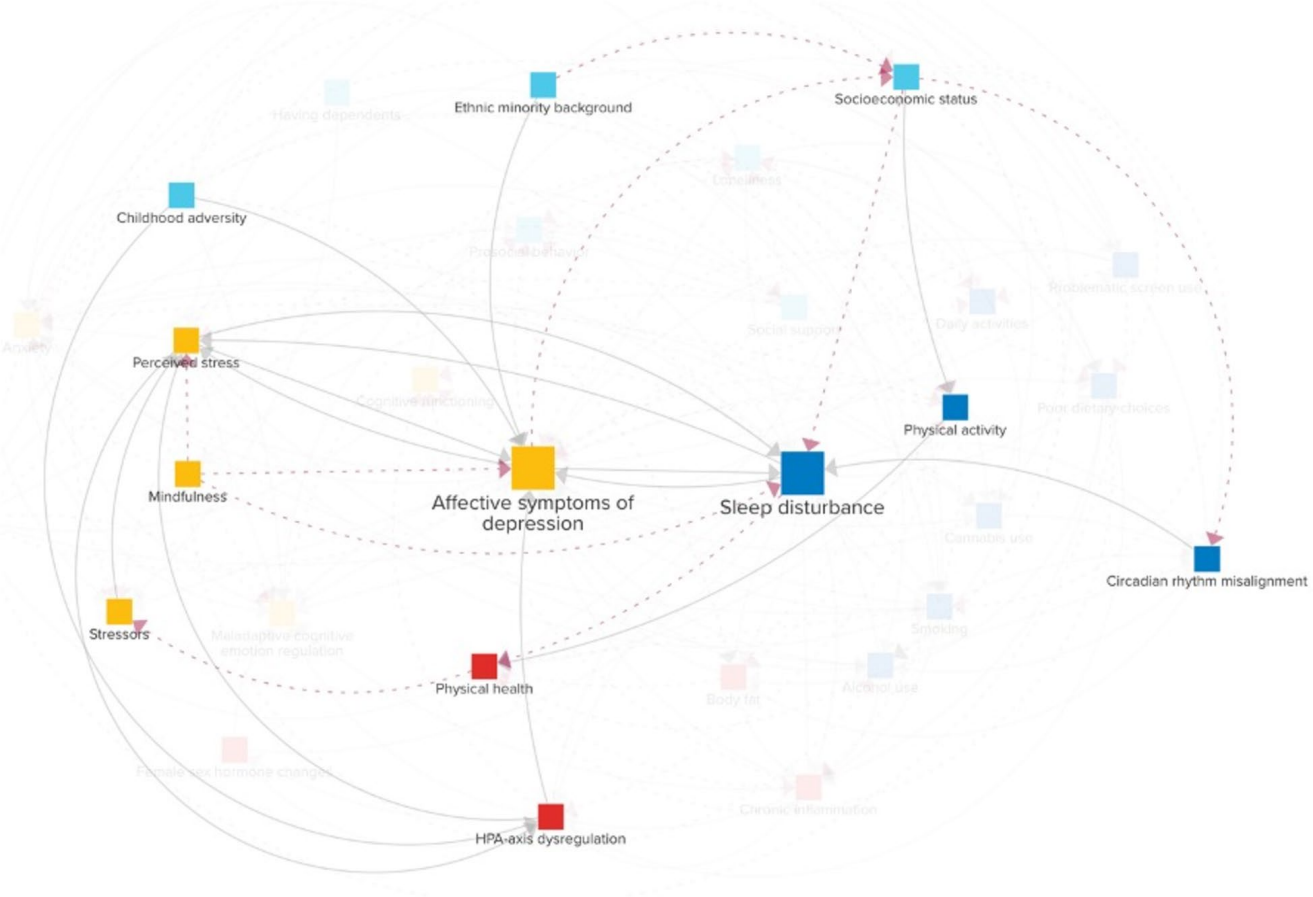
The relevant causal loop diagram pathways for a hypothetical case study of an immigrant exposed to high adversity in childhood (person B)

Figure 8 describes the paths relevant to person B, a male immigrant in his early thirties who experienced adverse conditions during his childhood. Traumatic events in his early life set the stage for chronic hyperactivity of his HPA-axis, leading to an exaggerated stress perception of daily challenges growing up. In later years, when starting his professional life, the persistent high stress level taxed his psychological and physiological resources, precipitating the development of *affective symptoms of depression*. These symptoms, in turn, heightened his sensitivity to stress, creating a vicious cycle where stress and affective depressive symptoms intensified each other, making daily life increasingly difficult to navigate.

Amidst these personal challenges, person B also faced hardships due to his *ethnic minority background*. He encountered discrimination that not only intensified his depressive symptoms but also impeded his occupational opportunities, putting him at a socioeconomic disadvantage. This disparity worsened as his mental health issues began to affect his work performance, which pushed him into lower-paying positions that required irregular hours or night shifts. These job conditions contributed to *circadian rhythm misalignment* and resultant sleep issues.

His reduced income further aggravated his sleep problems as it forced him into poorer living conditions, where environmental noise was rampant. His *physical health* also began to decline; Person B developed chronic back pain aggravated by a lack of access to affordable sports facilities, which restricted his *physical activity*. The toll on his health introduced additional stressors, which, in turn, amplified his stress perception. High levels of mental stress made it difficult for him to fall asleep and maintain deep restorative sleep, and inadequate sleep, in turn, heightened his *perceived stress*, forming a direct reinforcing feedback loop.

Fortunately, that same year, local authorities implemented a rent compensation program for low-income households. This significant relief from financial stress enabled person B to seek alternative, more fulfilling employment opportunities. At the same time, person B’s family doctor recommended a mindfulness-based stress reduction program, fully covered by basic health insurance, which began to gradually shift his perception of stress. Together, these changes helped person B manage his stress, easing both his affective depressive symptoms and sleep problems. Over time, these changes disrupted the vicious cycles that had dominated his recent years, paving the way toward stability and psychological well-being.

## Discussion

This paper introduced the Young Adult Sleep model, a CLD designed to examine the feedback mechanisms linking sleep disturbances and affective depressive symptoms in young adults. By integrating insights from two established CLD, findings from the literature, and input from 14 domain experts, the model offers an integrated view of the biological, psychological, behavioral, and social processes shaping mental health in this age group. The diagram illustrates numerous pathways through which sleep and mood problems can emerge and worsen, and may help understand individual trajectories (e.g., Figs. 7 and 8).

A key feature of the model is the dense network of reinforcing feedback loops, underscoring their role in driving system behavior [37, 45]. These loops may trap young adults in vicious cycles, such as those between *sleep disturbance*, *affective symptoms of depression*, and *smoking* (Fig. 4). As a result, variables that participate in many of these reinforcing loops (Fig. 3), including *anxiety*, *perceived stress*, *chronic inflammation*, *prosocial behavior*, and *loneliness*, might be influential drivers of *sleep disturbance* and *affective symptoms of depression*.

Many of the feedback loops span multiple domains, linking factors such as *loneliness*, *alcohol use*, *physical health*, and *well-being* (Fig. 5). Recognizing cross-scale loops may be particularly valuable, as such loops often go unnoticed when studies focus exclusively on single scientific domains (e.g., neurobiology) and isolate them from the broader influence of other important areas, such as the behavioral and social sciences. This can potentially lead to missed opportunities to identify new intervention targets. While existing studies support individual connections within these loops, their dynamic interplay may amplify effects or produce unexpected consequences. The CLD will, therefore, provide a basis for a data-driven system dynamics model, enabling empirical quantification and testing of intervention scenarios through simulation [35] by calibrating the model using multidimensional data from longitudinal cohort studies like the Healthy Brain Study [71].

The CLD is highly connected, with an average of 6 links per variable, which is higher than typically seen in similar diagrams. We interpret this level of connectedness as reflecting the complexity of mental health in young adults, justifying a comprehensive mapping rather than simplification, which could omit critical pathways. Our approach was intentionally inclusive: experts were encouraged to propose connections they considered plausible, and a connection was only removed if insufficiently supported by the literature or mechanistic explanations [35]. While this approach may have yielded less clarity about the most important or strongest connections, retaining all plausible links keeps the model open to data-driven refinement and allows subsequent computational modeling to determine whether certain connections might be negligible [35, 72, 73].

This study has several limitations. First, our asynchronous, assignment-based expert elicitation enabled broad geographical participation but lacked the interactive dialogue of traditional group model building [34, 37]. To address this, we used natural language processing to systematically analyze expert responses, integrated information from existing CLD, and iteratively refined the input across five assignments. Second, expert-driven methods inherently involve some subjectivity and may overrepresent particular areas of expertise [40]. To mitigate this, we assembled a diverse group with a range of experience in mental health and sleep research, as reflected in the relatively few links proposed multiple times. Third, the cited literature was not systematically reviewed due to the large number of connections, potentially omitting supporting evidence not familiar to the experts. Quality also varied, with many connections lacking support from randomized controlled trials or triangulation studies [74]. However, even where such evidence exists, it can rarely help distinguish direct from indirect effects, which we addressed by providing mechanistic explanations for each connection. Finally, although our approach was inclusive, experts may not have exhaustively considered all possible connections [35], so some may still be missing from the CLD.

Given these limitations, the Young Adult Sleep model should be viewed not as a final product but as a framework for synthesizing evidence on young adult mental health and supporting theory and hypothesis development [75]. The CLD is intended as a dynamic, iterative tool that the scientific community can continually refine and expand. To facilitate this iterative process, the interactive online version provides access to all underlying support materials and includes commenting functionality, enabling ongoing feedback and updates.

## Conclusions

We applied a complex systems thinking approach to better understand the dynamics of sleep problems and affective depressive symptoms in young adults. By integrating expert input, existing literature, and two existing CLD, we mapped a dense network of reinforcing loops spanning biological, psychological, behavioral, and social domains. The resulting Young Adult Sleep model highlights key variables driving these loops, balancing loops that may counteract them, and several cross-scale loops that may be overlooked in single-discipline studies. It also illustrates how multiple pathways may shape individual mental health trajectories. The model provides a foundation for hypothesis generation, data-driven system dynamics modeling, and in silico intervention testing, serving as a proof of concept for leveraging systems thinking to address the growing youth mental health crisis.

## Supplementary Information

Additional file 1: Figures S1–S2, Table S1: Figure S1—Workflow with the five assignmentsperformed by the domain expert group. Figure S2—Cosine similarities cut-off. Table S1—Mapping of variables from the CLD by Wittenborn et al. [1] to our CLD.

Additional file 2.

## Data Availability

Data sharing is not applicable to this article as no datasets were generated or analyzed during the current study. All generated materials are provided in Additional file 2.

## Abbreviations

CLD: Causal loop diagram
HPA: Hypothalamic-pituitary-adrenal
LLM: Large language model

## References

[1] McGorry PD, Mei C, Dalal N, Alvarez-Jimenez M, Blakemore S-J, Browne V, et al. The Lancet Psychiatry Commission on youth mental health. Lancet Psychiatry. 2024;11:731–74. 10.1016/S2215-0366(24)00163-9. 39147461 10.1016/S2215-0366(24)00163-9

[2] Arias D, Saxena S, Verguet S. Quantifying the global burden of mental disorders and their economic value. eClin Med. 2022. 10.1016/j.eclinm.2022.101675. 10.1016/j.eclinm.2022.101675 PMC9526145 36193171

[3] Goodwin RD, Dierker LC, Wu M, Galea S, Hoven CW, Weinberger AH. Trends in U.S. depression prevalence from 2015 to 2020: the widening treatment gap. Am J Prev Med. 2022;63:726. 10.1016/j.amepre.2022.05.014. 36272761 10.1016/j.amepre.2022.05.014 PMC9483000

[4] Rod NH, Davies M, de Vries TR, Kreshpaj B, Drews H, Nguyen T-L, et al. Young adulthood: a transitional period with lifelong implications for health and wellbeing. BMC Global and Public Health. 2025;3:25. 10.1186/s44263-025-00148-8. 40140902 10.1186/s44263-025-00148-8 PMC11948773

[5] McArdle N, Ward SV, Bucks RS, Maddison K, Smith A, Huang R-C, et al. The prevalence of common sleep disorders in young adults: a descriptive population-based study. Sleep. 2020;43:zsaa072. 10.1093/sleep/zsaa072. 32280974 10.1093/sleep/zsaa072

[6] Damgaard AJ, Sørensen JB, Jensen MM, Pedersen P. The association between sleep, mental health, and healthbehaviours: a Danish population-based cross-sectional study. Scand J Public Health. 2024;0(0). 10.1177/14034948241262366. 10.1177/14034948241262366 39129329

[7] Freeman D, Sheaves B, Waite F, Harvey AG, Harrison PJ. Sleep disturbance and psychiatric disorders. Lancet Psychiatry. 2020;7:628–37. 10.1016/S2215-0366(20)30136-X. 32563308 10.1016/S2215-0366(20)30136-X

[8] Huang B-H, del Pozo Cruz B, Teixeira-Pinto A, Cistulli PA, Stamatakis E. Influence of poor sleep on cardiovascular disease-free life expectancy: a multi-resource-based population cohort study. BMC Med. 2023;21:75. 10.1186/s12916-023-02732-x. 36859313 10.1186/s12916-023-02732-x PMC9979412

[9] Hare DL, Toukhsati SR, Johansson P, Jaarsma T. Depression and cardiovascular disease: a clinical review. Eur Heart J. 2014;35:1365–72. 10.1093/eurheartj/eht462. 24282187 10.1093/eurheartj/eht462

[10] Livingston G, Huntley J, Sommerlad A, Ames D, Ballard C, Banerjee S, et al. Dementia prevention, intervention, and care: 2020 report of the Lancet Commission. Lancet. 2020;396:413–46. 10.1016/S0140-6736(20)30367-6. 32738937 10.1016/S0140-6736(20)30367-6 PMC7392084

[11] Bubu OM, Brannick M, Mortimer J, Umasabor-Bubu O, Sebastião YV, Wen Y, et al. Sleep, Cognitive impairment, and Alzheimer’s disease: A Systematic Review and Meta-Analysis, Sleep. 2017;40(1):zsw032. 10.1093/sleep/zsw032. 10.1093/sleep/zsw032 28364458

[12] Uleman JF, Melis RJ, Ntanasi E, Scarmeas N, Hoekstra AG, Quax R, et al. Simulating the multicausality of Alzheimer’s disease with system dynamics. Alzheimers Dement. 2023. 10.1002/alz.12923. 36794757 10.1002/alz.12923

[13] Jin Q, Yang N, Dai J, Zhao Y, Zhang X, Yin J, et al. Association of sleep duration with all-cause and cardiovascular mortality: a prospective cohort study. Front Public Health. 2022. 10.3389/fpubh.2022.880276. 35910926 10.3389/fpubh.2022.880276 PMC9334887

[14] Walker ER, McGee RE, Druss BG. Mortality in mental disorders and global disease burden implications. JAMA Psychiatr. 2015;72:334–41. 10.1001/jamapsychiatry.2014.2502. 10.1001/jamapsychiatry.2014.2502 PMC4461039 25671328

[15] Riemann D, Benz F, Dressle RJ, Espie CA, Johann AF, Blanken TF, et al. Insomnia disorder: state of the science and challenges for the future. J Sleep Res. 2022;31:e13604. 10.1111/jsr.13604. 35460140 10.1111/jsr.13604

[16] Rosenström T, Jokela M, Puttonen S, Hintsanen M, Pulkki-Råback L, Viikari JS, et al. Pairwise measures of causal direction in the epidemiology of sleep problems and depression. PLoS ONE. 2012;7:e50841. 10.1371/journal.pone.0050841. 23226400 10.1371/journal.pone.0050841 PMC3511346

[17] Otte Andersen T, Skovlund Dissing A, Rosenbek Severinsen E, Kryger Jensen A, Thanh Pham V, Varga TV, et al. Predicting stress and depressive symptoms using high-resolution smartphone data and sleep behavior in Danish adults. Sleep. 2022;45:zsac067. 10.1093/sleep/zsac067. 35298650 10.1093/sleep/zsac067

[18] Riemann D, Berger M, Voderholzer U. Sleep and depression — results from psychobiological studies: an overview. Biol Psychol. 2001;57:67–103. 10.1016/S0301-0511(01)00090-4. 11454435 10.1016/s0301-0511(01)00090-4

[19] Andersen TO, Sejling C, Jensen AK, Drews HJ, Ritz B, Varga TV, et al. Nighttime smartphone use, sleep quality, and mental health: investigating a complex relationship. Sleep. 2023. 10.1093/sleep/zsad256. 37758231 10.1093/sleep/zsad256

[20] Hachenberger J, Ten Brink M, Kerkhoff D, Baron S, Schabus M, Lemola S. Exploring the bidirectional within-subject relationship between sleep and affective wellbeing: insights from an intensive longitudinal study. Int J Clin Health Psychol. 2025;25:100648. 10.1016/j.ijchp.2025.100648. 41322973 10.1016/j.ijchp.2025.100648 PMC12662039

[21] Wittenborn AK, Rahmandad H, Rick J, Hosseinichimeh N. Depression as a systemic syndrome: mapping the feedback loops of major depressive disorder. Psychol Med. 2016;46:551–62. 26621339 10.1017/S0033291715002044 PMC4737091

[22] van der Wal JM, Van Borkulo CD, Deserno M, Breedvelt JJF, Lees M, Lokman C, et al. Advancing urban mental health research: from complexity to actionable targets for intervention. Lancet Psychiatry. 2021:1–10. 10.1016/S2215-0366(21)00047-X. 10.1016/S2215-0366(21)00047-X 34627532

[23] Cramer AOJ, van Borkulo CD, Giltay EJ, van der Maas HLJ, Kendler KS, Scheffer M, et al. Major depression as a complex dynamic system. PLoS ONE. 2016;11:e0167490. 10.1371/journal.pone.0167490. 27930698 10.1371/journal.pone.0167490 PMC5145163

[24] Olthof M, Hasselman F, Maatman FO, Bosman A, Lichtwarck-Aschoff A. Complexity theory of psychopathology. 2020. 10.31234/osf.io/f68ej. 10.1037/abn0000740 37126062

[25] Rod NH, Broadbent A, Rod MH, Russo F, Arah OA, Stronks K. Complexity in epidemiology and public health addressing complex health problems through a mix of epidemiologic methods and data. Epidemiology. 2023. 10.1097/EDE.0000000000001612. 37042967 10.1097/EDE.0000000000001612 PMC10712344

[26] Galea S, Riddle M, Kaplan GA. Causal thinking and complex system approaches in epidemiology. Int J Epidemiol. 2010;39:97–106. 10.1093/ije/dyp296. 19820105 10.1093/ije/dyp296 PMC2912489

[27] Diez Roux AV. Complex systems thinking and current impasses in health disparities research. Am J Public Health. 2011;101:1627–34. 10.2105/AJPH.2011.300149. 21778505 10.2105/AJPH.2011.300149 PMC3154209

[28] Rutter H, Savona N, Glonti K, Bibby J, Cummins S, Finegood DT, et al. The need for a complex systems model of evidence for public health. Lancet. 2017;390:2602–4. 10.1016/S0140-6736(17)31267-9. 28622953 10.1016/S0140-6736(17)31267-9

[29] Borsboom D. A network theory of mental disorders. World Psychiatry. 2017;16:5–13. 28127906 10.1002/wps.20375 PMC5269502

[30] Robinaugh DJ, Hoekstra RH, Toner ER, Borsboom D. The network approach to psychopathology: a review of the literature 2008–2018 and an agenda for future research. Psychol Med. 2020;50:353–66. 31875792 10.1017/S0033291719003404 PMC7334828

[31] Reesen JE, Hoogendoorn AW, Leerssen J, Lancee J, Blanken TF, Batelaan NM, et al. A call for transdiagnostic attention to insomnia and its treatment in mental healthcare. J Sleep Res. 2024;33:e14049. 10.1111/jsr.14049. 38351526 10.1111/jsr.14049

[32] Lunansky G, Hoekstra RHA, Blanken TF. Disentangling the role of affect in the evolution of depressive complaints using complex dynamical networks. Collabra: Psychology. 2023;9:74841. 10.1525/collabra.74841.

[33] Blanken TF, Borsboom D, Penninx BW, Van Someren EJ. Network outcome analysis identifies difficulty initiating sleep as a primary target for prevention of depression: a 6-year prospective study. Sleep. 2020;43:zsz288. 10.1093/sleep/zsz288. 31789381 10.1093/sleep/zsz288 PMC7215262

[34] Uleman JF, Melis RJ, Quax R, van der Zee EA, Thijssen D, Dresler M, et al. Mapping the multicausality of Alzheimer’s disease through group model building. GeroScience. 2020:1–15. 10.1007/s11357-020-00228-7. 10.1007/s11357-020-00228-7 PMC8110634 32780293

[35] Crielaard L, Uleman JF, Chatel BDL, Epskamp S, Sloot PMA, Quax R. Refining the causal loop diagram: a tutorial for maximizing the contribution of domain expertise in computational system dynamics modeling. Psychol Methods. 2024;29(1):169-201. 10.1037/met0000484. 10.1037/met0000484 35549316

[36] Kenzie ES, Parks EL, Bigler ED, Wright DW, Lim MM, Chesnutt JC, et al. The dynamics of concussion: mapping pathophysiology, persistence, and recovery with causal-loop diagramming. Front Neurol. 2018;9:203. 29670568 10.3389/fneur.2018.00203 PMC5893805

[37] Uleman JF, Stronks K, Rutter H, Arah OA, Rod NH. Mapping complex public health problems with causal loop diagrams. Int J Epidemiol. 2024. 10.1093/ije/dyae091. 38990180 10.1093/ije/dyae091 PMC13377455

[38] Crielaard L, Dutta P, Quax R, Nicolaou M, Merabet N, Stronks K, et al. Social norms and obesity prevalence: from cohort to system dynamics models. Obes Rev. 2020. 10.1111/obr.13044. 32400030 10.1111/obr.13044 PMC7507199

[39] Hosseinichimeh N, Wittenborn AK, Rick J, Jalali MS, Rahmandad H. Modeling and estimating the feedback mechanisms among depression, rumination, and stressors in adolescents. PLoS ONE. 2018;13:e0204389. 30261010 10.1371/journal.pone.0204389 PMC6160072

[40] Uleman JF, Luijten M, Abdo WF, Vyrastekova J, Gerhardus A, Runge J, et al. Triangulation for causal loop diagrams: constructing biopsychosocial models using group model building, literature review, and causal discovery. npj Complex. 2024;1:1–11. 10.1038/s44260-024-00017-9.

[41] Crielaard L, Nicolaou M, Sawyer A, Quax R, Stronks K. Understanding the impact of exposure to adverse socioeconomic conditions on chronic stress from a complexity science perspective. BMC Med. 2021;19:242. 10.1186/s12916-021-02106-1. 34635083 10.1186/s12916-021-02106-1 PMC8507143

[42] Barsties LS, van den Berg SW, Leone SS, Nicolaou M, van Oostrom SH. A system science perspective on burn-out: development of an expert-based causal loop diagram. Front Public Health. 2023. 10.3389/fpubh.2023.1271591. 38035310 10.3389/fpubh.2023.1271591 PMC10687398

[43] van der Wal JM, Huth KBS, Lok A, Bockting CL, Stronks K, Nicolaou M. Exploring the mechanisms underlying increased risk of depressive disorder in ethnic minority populations in Europe: a causal loop diagram. Soc Sci Med. 2024;351:116977. 10.1016/j.socscimed.2024.116977. 38788426 10.1016/j.socscimed.2024.116977

[44] Richardson GP. Problems with causal-loop diagrams. Syst Dyn Rev. 1986;2:158–70. 10.1002/sdr.4260020207.

[45] Sterman J. Business dynamics: systems thinking and modeling for a complex world. Irwin/McGraw-Hill; 2000.

[46] Pennington J, Socher R, Manning C. GloVe: Global vectors for word representation. In: Moschitti A, Pang B, Daelemans W, editors. Proceedings of the 2014 Conference on Empirical Methods in Natural Language Processing (EMNLP). Doha, Qatar: Association for Computational Linguistics; 2014. p. 1532–43. 10.3115/v1/D14-1162.

[47] Řehůřek R, Sojka P, others. Gensim—statistical semantics in python. Retrieved from genism org. 2011.

[48] Reimers N, Gurevych I. Making monolingual sentence embeddings multilingual using knowledge distillation. In: Proceedings of the 2020 Conference on Empirical Methods in Natural Language Processing. Association for Computational Linguistics; 2020.

[49] Valdivia Cabrera M, Johnstone M, Hayward J, Bolton KA, Creighton D. Integration of large-scale community-developed causal loop diagrams: a Natural Language Processing approach to merging factors based on semantic similarity. BMC Public Health. 2025;25:923. 10.1186/s12889-025-22142-3. 40055777 10.1186/s12889-025-22142-3 PMC11889750

[50] Reimers N, Gurevych I. Sentence-BERT: sentence embeddings using siamese BERT-networks. In: Proceedings of the 2019 Conference on Empirical Methods in Natural Language Processing. Association for Computational Linguistics; 2019.

[51] Pedregosa F, Varoquaux G, Gramfort A, Michel V, Thirion B, Grisel O, et al. Scikit-learn: machine learning in Python. J Mach Learn Res. 2011;12:2825–30.

[52] Albrecht J. Finding a “kneedle” in a haystack: detecting knee points in system behavior. cs.williams.edu.

[53] Thapar A, Eyre O, Patel V, Brent D. Depression in young people. Lancet. 2022;400:617–31. 10.1016/S0140-6736(22)01012-1. 35940184 10.1016/S0140-6736(22)01012-1

[54] Gao J, Ding X, Qin B, Liu T. Is ChatGPT a good causal reasoner? A comprehensive evaluation. In: Bouamor H, Pino J, Bali K, editors. Findings of the Association for Computational Linguistics: EMNLP 2023. Singapore: Association for Computational Linguistics; 2023. p. 11111–26. 10.18653/v1/2023.findings-emnlp.743.

[55] Akhavan A, Jalali MS. Generative AI and simulation modeling: how should you (not) use large language models like ChatGPT. Syst Dyn Rev. 2024;40:e1773. 10.1002/sdr.1773.

[56] Wimmelmann CL, Sejling C, Clarke RB, Elsenburg LK, Sørensen TIA, Rod NH. Childhood adversity trajectories and weight status in young adult men: a register-based study including 359,783 Danish men. Int J Obes. 2024;48:1157–63. 10.1038/s41366-024-01540-4. 10.1038/s41366-024-01540-4 PMC11281903 38816565

[57] van Amsterdam JGC, Benschop A, van Binnendijk S, Snijder MB, Lok A, Schene AH, et al. A comparison of excessive drinking, binge drinking and alcohol dependence in ethnic minority groups in the Netherlands: The HELIUS Study. Eur Addict Res. 2020;26:66–76. 10.1159/000504881. 31812961 10.1159/000504881 PMC7114898

[58] Pascoe EA, Smart Richman L. Perceived discrimination and health: a meta-analytic review. Psychol Bull. 2009;135:531–54. 10.1037/a0016059. 19586161 10.1037/a0016059 PMC2747726

[59] Cover TM, Thomas JA. Elements of information theory 2nd edition (Wiley series in telecommunications and signal processing). Wiley-Interscience; 2006.

[60] Treur JL, Munafò MR, Logtenberg E, Wiers RW, Verweij KJH. Using Mendelian randomization analysis to better understand the relationship between mental health and substance use: a systematic review. Psychol Med. 2021;51:1593–624. 10.1017/S003329172100180X. 34030749 10.1017/S003329172100180X PMC8327626

[61] Fang H, Tu S, Sheng J, Shao A. Depression in sleep disturbance: a review on a bidirectional relationship, mechanisms and treatment. J Cell Mol Med. 2019;23:2324–32. 10.1111/jcmm.14170. 30734486 10.1111/jcmm.14170 PMC6433686

[62] Hamidovic A, de Wit H. Sleep deprivation increases cigarette smoking. Pharmacol Biochem Behav. 2009;93:263–9. 10.1016/j.pbb.2008.12.005. 19133287 10.1016/j.pbb.2008.12.005 PMC2706278

[63] Dugas EN, Sylvestre MP, O’Loughlin EK, Brunet J, Kakinami L, Constantin E, et al. Nicotine dependence and sleep quality in young adults. Addict Behav. 2017;65:154–60. 10.1016/j.addbeh.2016.10.020. 27816041 10.1016/j.addbeh.2016.10.020

[64] Franzen PL, Buysse DJ. Sleep disturbances and depression: risk relationships for subsequent depression and therapeutic implications. Dialogues Clin Neurosci. 2008;10:473–81. 10.31887/DCNS.2008.10.4/plfranzen. 19170404 10.31887/DCNS.2008.10.4/plfranzen PMC3108260

[65] Forrester JW. Urban dynamics. M.I.T. Press; 1969.

[66] Bohlmeijer E, Jacobs N, Walburg J, Westerhof G, Walburg JA. Handboek positieve psychologie: theorie, onderzoek en interventies. Boom; 2021.

[67] Brethel-Haurwitz KM, Stoianova M, Marsh AA. Empathic emotion regulation in prosocial behaviour and altruism. Cogn Emot. 2020;34:1532–48. 10.1080/02699931.2020.1783517. 32576078 10.1080/02699931.2020.1783517

[68] Chee PX, Shimshock CJ, Le BM. Prosociality as a means to buffer loneliness and strengthen well-being. J Soc Pers Relat. 2025;42:2619–40. 10.1177/02654075251344817.

[69] Wootton RE, Greenstone HSR, Abdellaoui A, Denys D, Verweij KJH, Munafò MR, et al. Bidirectional effects between loneliness, smoking and alcohol use: evidence from a Mendelian randomization study. Addiction. 2021;116:400–6. 10.1111/add.15142. 32542815 10.1111/add.15142

[70] Li M, Zhang X, Chen K, Miao Y, Xu Y, Sun Y, et al. Alcohol exposure and disease associations: a Mendelian randomization and meta-analysis on weekly consumption and problematic drinking. Nutrients. 2024. 10.3390/nu16101517. 38794754 10.3390/nu16101517 PMC11123792

[71] Consortium HBS, Aarts E, Akkerman A, Altgassen M, Bartels R, Beckers D, et al. Protocol of the Healthy Brain Study: an accessible resource for understanding the human brain and how it dynamically and individually operates in its bio-social context. PLoS ONE. 2021;16:e0260952. 10.1371/journal.pone.0260952. 34965252 10.1371/journal.pone.0260952 PMC8716054

[72] Brunton SL, Proctor JL, Kutz JN. Discovering governing equations from data by sparse identification of nonlinear dynamical systems. Proc Natl Acad Sci U S A. 2016;113:3932–7. 10.1073/pnas.1517384113. 27035946 10.1073/pnas.1517384113 PMC4839439

[73] Uleman JF, Melis RJ, Hoekstra AG, Rikkert MGO, Quax R. Exploring the potential impact of multi-factor precision interventions in Alzheimer’s disease with system dynamics. J Biomedical Inform. 2023;145:104462. 10.1016/j.jbi.2023.104462. 10.1016/j.jbi.2023.104462 37516375

[74] Lawlor DA, Tilling K, Davey Smith G. Triangulation in aetiological epidemiology. Int J Epidemiol. 2016;45:1866–86. 10.1093/ije/dyw314. 28108528 10.1093/ije/dyw314 PMC5841843

[75] Uleman JF, Quax R, Melis RJF, Hoekstra AG, Olde Rikkert MGM. The need for systems thinking to advance Alzheimer’s disease research. Psychiatr Res. 2024;333:115741. 10.1016/j.psychres.2024.115741. 10.1016/j.psychres.2024.115741 38277813

