# Supplementary material for: The Young Adult Sleep model: an evolving causal loop diagram of mental health dynamics
Source: BMC Med. 2026 Mar 16;24:159. doi: 10.1186/s12916-026-04738-7 (PMC12990402; doi:10.1186/s12916-026-04738-7)
Supplement: Supplementary file 1 — Additional file 1: Figures S1–S2, Table S1: Figure S1—Workflow with the five assignmentsperformed by the domain expert group. Figure S2—Cosine similarities cut-off. Table S1—Mapping of variables from the CLD by Wittenborn et al. [1] to our CLD. [file 12916_2026_4738_MOESM1_ESM.pdf]

Additional file 1

Assignment workflow

Our assignment workflow is summarized in Figure S1.

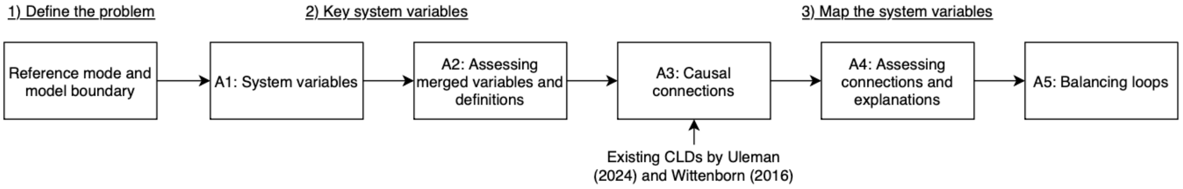

Figure S1 *Workflow with the five assignments (A1-A5) performed by the domain expert group*

Variables proposed by the domain experts in Assignment 1

The unique variables suggested by the domain experts in alphabetic order are: (Childhood) trauma / stressful life events, Accessibility to mental and general health care, Accidents, Addiction, ADHD, Alcohol (mis)use, Alcohol consumption, Alcohol intake, Alcohol use, Amygdala function, Anxiety, Appetite, Bad lifestyle choices, Belongingness, Biological gender, Biological stress, BMI, Body mass index, Brain inflammation, BDNF genes, Cannabis (mis)use, Care for children, Caregiving of newborns, Childhoood adversity, Cigarette smoking, Circadian rhythm disruption, e.g. shift work, Chronic pain, Chronic somatic disease, Cognitive abilities, Cognitive biases, Cognitive impairment, Cortisol, Daily activities, Daily hassles, Default mode network activation, Delayed sleep phase, Depression, Discrimination, Drug use, Education/Income, Emotion regulation, Emotional stress, Employment, Endocrine system dysfunction, Environmental factors, Ethnic background, Exercise/physical activity, Excessive alcohol intake, Exposure to daylight, External stressors, Friendship quality, Gender differences, General sense of wellbeing, Genetic liability, Genetic predisposition, Glucocorticoid receptor upregulation and HPA axis hyperactivity, Gut microbiota disruption/intestinal permeability, Having dependents, High blood pressure, Hippocampal function, Hormonal changes, HPA axis activity, HPA-Axis, Illicit drug use, Immune dysregulation, Immune system /Common infections, Infections/inflammation, Increased risk for common infections, Increased sympathetic drive, Life events, Living situation, Living with a partner, Loneliness, Loneliness social withdrawal, Low-grade systemic inflammation, Medication, Memory function, Mental health disorder, Metabolic dysregulation, Metabolism, Mindfulness, Negative affect, Negative life events, Night

shifts at work, Nighttime smartphone use, Obesity, Online environment/behaviour, Oxytocin genes, Perceived social inclusion, Perceived social support, Perceived stress, Personality traits, Peripheral immune function, Physical activity, Physical exercise, Physical inactivity, Physiological stress response, Poor diet, Positive affect, Positive emotions, Prefrontal cortex function, Problems with social interactions, Prosocial behavior, PTSD, QoL, Reduced activity levels, Resilience, Rumination, Rumination/arousal, Salience network activation, Screen time, Severe somatic disease, Sex hormones, Sex steroid hormones/ Pregnancy, Sexual dysfunction, Sleep apnea, Sleep duration, Sleep fragmentation, Sleep hygiene, Sleep regularity, Sleep routines, Sleep stability, Smoking, Social activities, Social behavior/isolation, Social contact, Social functioning, Social interactions, Social jetlag, Social media / screen use, Social media use, Social media use/screen time, Social network (friends/family), Social status, Socio-economic position, Socio-economic status, Socioeconomic hardship / status, Socioeconomic status, Societal expectations, Study/work/social stress, Substance use, Synaptic pruning, Temperature, Time preference/present bias/time self-continuity, Traumatic experiences, Undiagnosed sleep disorders, Well-being.

Exact duplicates proposed by the experts with the number of times they were proposed:

Alcohol use: 3, Anxiety: 4, Chronic pain: 2, Cortisol: 4, Emotion regulation: 2, Exercise/physical activity: 2, Genetic liability: 2, Inflammation: 2, Life events: 2, Loneliness: 3, Medication: 2, Perceived social support: 2, Physical activity: 4, Rumination: 4, Smoking: 4, Social jetlag: 2, Social support: 2

Variable merging with natural language processing

Cosine similarity cut-off:

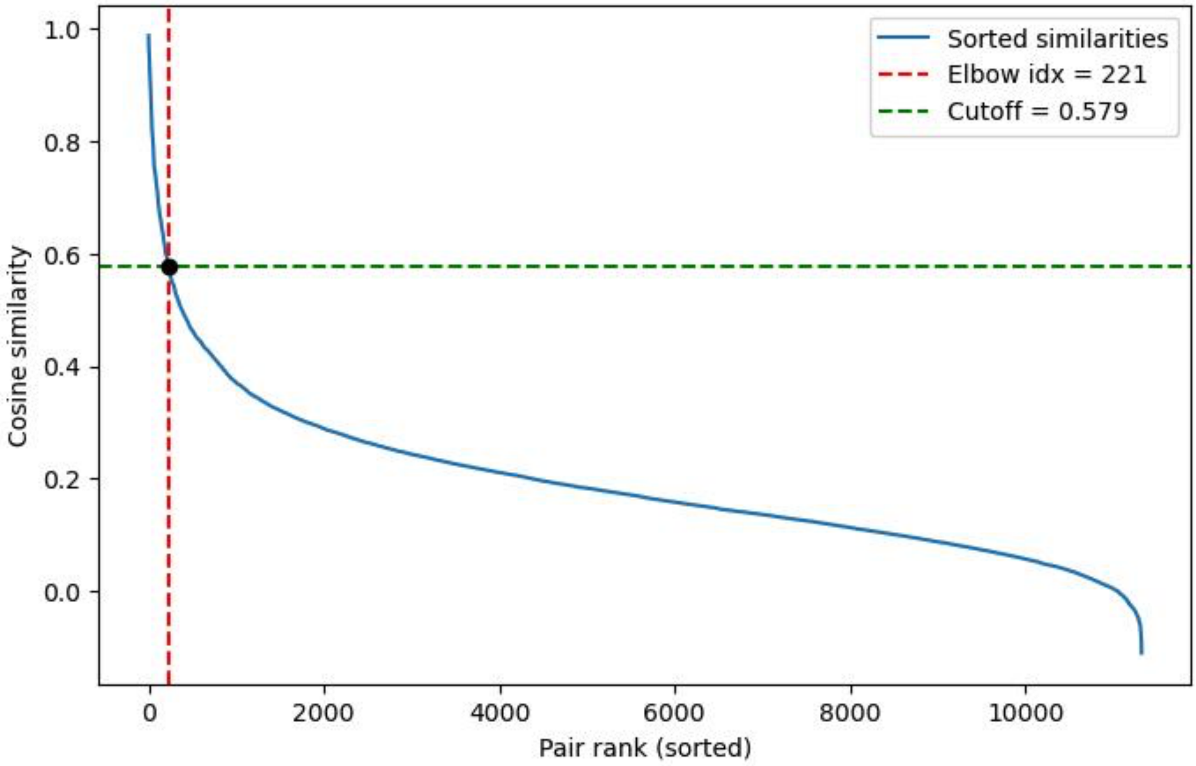

Figure S2 *Cosine similarities cut-off*

Clusters:

Below we provide the identified clusters and how they related to the selected CLD variables. Each CLD variable can be mentioned multiple times and there can also be multiple variables per cluster.

Maladaptive cognitive emotion regulation

Cluster 27 (Total Rating = 13.0):

Rumination | Ratings: [3.0, 2.0, 3.0, 2.0]

Rumination/arousal | Ratings: [3.0]

Wellbeing

Cluster 63 (Total Rating = 3.0):

QoL | Ratings: [3.0]

Cluster 23 (Total Rating = 5.0):

General sense of wellbeing | Ratings: [2.0]

Well-being | Ratings: [3.0]

Smoking

Cluster 26 (Total Rating = 9.0):

Cigarette smoking | Ratings: [3.0]

Smoking | Ratings: [2.0, 2.0, 1.0, 1.0]

Socioeconomic status

Cluster 0 (Total Rating = 16.0):

Socioeconomic status | Ratings: [3.0]

Social status | Ratings: [3.0]

Socio-economic status | Ratings: [3.0]

Socio-economic position | Ratings: [3.0]

Socioeconomic hardship / status | Ratings: [3.0]

Societal expectations | Ratings: [1.0]

Loneliness

Cluster 13 (Total Rating = 10.0):

Loneliness | Ratings: [3.0, 1.0, 3.0]

Loneliness social withdrawal | Ratings: [3.0]

Cluster 46 (Total Rating = 3.0):

Belongingness | Ratings: [3.0]

Anxiety

Cluster 64 (Total Rating = 10.0):

Anxiety | Ratings: [3.0, 2.0, 2.0, 3.0]

Cannabis use, Alcohol use, Physical health

Cluster 2 (Total Rating = 13.0):

Illicit drug use | Ratings: [2.0]

Addiction | Ratings: [2.0]

Medication | Ratings: [1.0, 1.0]

Cannabis (mis)use | Ratings: [2.0]

Drug use | Ratings: [3.0]

Substance use | Ratings: [2.0]

Affective symptoms of depression

Cluster 7 (Total Rating = 8.0):

Positive emotions | Ratings: [3.0]

Emotion regulation | Ratings: [2.0, 3.0]

Cluster 28 (Total Rating = 5.0):

Negative affect | Ratings: [3.0]

Positive affect | Ratings: [2.0]

Ethnic minority background

Cluster 67 (Total Rating = 3.0):

Discrimination | Ratings: [3.0]

Problematic screen use

Cluster 31 (Total Rating = 3.0):

Online environment/behaviour | Ratings: [3.0]

Cluster 38 (Total Rating = 4.0):

Social media / screen use | Ratings: [1.0]

Social media use | Ratings: [1.0]

Social media use/screen time | Ratings: [2.0]

Cluster 80 (Total Rating = 3.0):

Nighttime smartphone use | Ratings: [3.0]

Cognitive functioning

Cluster 11 (Total Rating = 6.0):

Cognitive abilities | Ratings: [2.0]

Cognitive biases | Ratings: [2.0]

Cognitive impairment | Ratings: [2.0]

Social support, Prosocial behavior, Daily activities

Cluster 9 (Total Rating = 17.0):

Social interactions | Ratings: [3.0]

Social behavior/isolation | Ratings: [2.0]

Social functioning | Ratings: [3.0]

Problems with social interactions | Ratings: [1.0]

Prosocial behavior | Ratings: [3.0]

Social contact | Ratings: [3.0]

Social activities | Ratings: [2.0]

Ethnic minority background

Cluster 44 (Total Rating = 3.0):

Ethnic background | Ratings: [3.0]

Sleep disturbance

Cluster 4 (Total Rating = 16.0):

Sleep fragmentation | Ratings: [2.0]

Sleep duration | Ratings: [2.0]

Delayed sleep phase | Ratings: [2.0]

Sleep apnea | Ratings: [1.0]

Sleep stability | Ratings: [2.0]

Sleep routines | Ratings: [3.0]

Sleep hygiene | Ratings: [2.0]

Sleep regularity | Ratings: [1.0]

Undiagnosed sleep disorders | Ratings: [1.0]

Stressors, Daily activities

Cluster 8 (Total Rating = 5.0):

Daily hassles | Ratings: [3.0]

Daily activities | Ratings: [2.0]

Circadian rhythm misalignment

Cluster 49 (Total Rating = 4.0):

Social jetlag | Ratings: [2.0, 2.0]

Cluster 52 (Total Rating = 3.0):

Night shifts at work | Ratings: [3.0]

Female sex hormone changes

Cluster 18 (Total Rating = 6.0):

Sex steroid hormones/ Pregnancy | Ratings: [2.0]

Hormonal changes | Ratings: [2.0]

Sex hormones | Ratings: [2.0]

Physical health

Cluster 29 (Total Rating = 3.0):

Metabolism | Ratings: [2.0]

Metabolic dysregulation | Ratings: [1.0]

Cluster 12 (Total Rating = 4.0):

Severe somatic disease | Ratings: [2.0]

Chronic somatic disease | Ratings: [2.0]

Cluster 40 (Total Rating = 4.0):

Chronic pain | Ratings: [3.0, 1.0]

Social support

Cluster 1 (Total Rating = 13.0):

Perceived social support | Ratings: [3.0, 3.0]

Social support | Ratings: [2.0, 3.0]

Perceived social inclusion | Ratings: [2.0]

Chronic low-grade inflammation

Cluster 17 (Total Rating = 12.0):

Infections/inflammation | Ratings: [2.0]

Inflammation | Ratings: [2.0, 2.0]

Low-grade systemic inflammation | Ratings: [3.0]

Immune system /Common infections | Ratings: [3.0]

Having dependents

Cluster 6 (Total Rating = 5.0):

Living with a partner | Ratings: [2.0]

Having dependents | Ratings: [3.0]

Cluster 36 (Total Rating = 5.0):

Care for children | Ratings: [2.0]

Caregiving of newborns | Ratings: [3.0]

Cluster 33 (Total Rating = 3.0):

Young children (0-3years) | Ratings: [3.0]

Body fat

Cluster 5 (Total Rating = 7.0):

BMI | Ratings: [2.0]

Body mass index | Ratings: [3.0]

Obesity | Ratings: [2.0]

Perceived stress, Stressors, HPA-axis dysregulation

Cluster 19 (Total Rating = 20.0):

Perceived stress | Ratings: [3.0]

Biological stress | Ratings: [3.0]

Physiological stress response | Ratings: [3.0]

External stressors | Ratings: [2.0]

Study/work/social stress | Ratings: [3.0]

Work stress | Ratings: [3.0]

Emotional stress | Ratings: [3.0]

Poor dietary choices

Cluster 61 (Total Rating = 3.0):

Poor diet | Ratings: [3.0]

Alcohol use

Cluster 16 (Total Rating = 15.0):

Alcohol (mis)use | Ratings: [3.0]

Alcohol consumption | Ratings: [1.0]

Excessive alcohol intake | Ratings: [3.0]

Alcohol use | Ratings: [2.0, 2.0, 2.0]

Alcohol intake | Ratings: [2.0]

Chronic low-grade inflammation

Cluster 30 (Total Rating = 5.0):

Immune dysregulation | Ratings: [3.0]

Peripheral immune function | Ratings: [2.0]

HPA-axis dysregulation

Cluster 34 (Total Rating = 5.0):

HPA-Axis | Ratings: [3.0]

HPA axis activity | Ratings: [2.0]

Cluster 47 (Total Rating = 12.0):

Cortisol | Ratings: [3.0, 3.0, 3.0, 3.0]

Physical activity

Cluster 20 (Total Rating = 21.0):

Physical exercise | Ratings: [3.0]

Exercise/physical activity | Ratings: [2.0, 2.0]

Physical activity | Ratings: [2.0, 3.0, 3.0, 3.0]

Physical inactivity | Ratings: [3.0]

Cluster 70 (Total Rating = 3.0):

Reduced activity levels | Ratings: [3.0]

Childhood adversity

Cluster 3 (Total Rating = 7.0):

Traumatic experiences | Ratings: [3.0]

(Childhood) trauma / stressful life events | Ratings: [3.0]

Childhood adversity | Ratings: [1.0]

Cluster 14 (Total Rating = 9.0):

Negative life events | Ratings: [3.0]

Life events | Ratings: [3.0, 3.0]

Chronic inflammation

Cluster 53 (Total Rating = 3.0):

Brain inflammation | Ratings: [3.0]

Mapping variables from Wittenborn et al. [1] into our CLD

Before incorporating connections from the existing CLDs, we first applied a CLD simplification procedure [2] to the CLD by Wittenborn et al. [1]. Our simplification process entailed replacing variables with a single outgoing connection by direct connections from their cause(s) to their effect. Consequently, we replaced "Inhibitory effect of glucocorticoid on cytokine," "Feedback inhibition of HPA," "Chronic medical illness," "Biased attention and processing," "Deficiency of working memory," and "Encoding of negative thoughts" with direct causal connections. We then combined the variables "Negative cognitive representations" and "Negative affect, interpretation, and processing" into a broader construct termed *Affective symptoms of depression,* and integrated the variables "Learning, reward, processing, and memory" and "Cognitive performance" into a single variable called *Cognitive functioning*. We also combined "Cortisol" and "Effective GR" into HPA-axis dysfunction. Finally, one variable from Wittenborn et al. [1], "Dysfunctional behaviors," was incorporated into three variables, namely *Smoking*, *Physical activity*, and *Prosocial behavior*, based on the provided references to the scientific literature. This mapping is summarized in Table D1. Note that some of our CLD variables are broader than Wittenborn's; for instance, *Maladaptive cognitive emotion regulation* encompasses "Rumination" but also other types of strategies like self-blame and catastrophizing. All suggested connections were assessed by the domain expert group and retained only if all agreed to their inclusion.

**Wittenborn et al. [1] CLD's variables**	**Our CLD's variables**
Sleep problem	Sleep disturbance

Negative cognitive representations; Negative affect, interpretation, and processing	Affective symptoms of depression
Interpersonal relationship quality	Social support
Rumination	Maladaptive cognitive emotion regulation
Physical inactivity	Physical activity
Physical health	Physical health
Dysfunctional behaviors	Prosocial behavior; Smoking; Physical activity
Cognitive performance; Learning, reward processing, and memory	Cognitive functioning
Perceived stress	Perceived stress
Cortisol; Effective GR	HPA-axis dysregulation
Cytokine	Chronic inflammation
Economic status	Socioeconomic status
Stress stimuli	Stressors

Table S1: Mapping of variables from the CLD by Wittenborn et al. [1] to our CLD

References

1. Wittenborn AK, Rahmandad H, Rick J, Hosseinichimeh N. Depression as a systemic syndrome: mapping the feedback loops of major depressive disorder. Psychol Med. 2016;46:551–62.

2. Bureš V. A method for simplification of complex group causal loop diagrams based on endogenisation, encapsulation and order-oriented reduction. Systems. 2017;5:46.
